# Self-administration of Recombinant Human Growth Hormone with an Electronic Device: Clinical, Economic and Management Benefits of Objective Adherence Monitoring

**Published:** 2015-12-07

**Authors:** Zuleika Saz-Parkinson, Maria Del Sol Granados Alonso, Carmen Bouza, José Luis Poveda Andrés, José María Amate

**Affiliations:** 1 Instituto de Salud Carlos III, Agencia de Evaluación de Tecnologías Sanitarias, Madrid, Spain; 2 Servicio de Medicina Preventiva y Salud Pública, Hospital Universitario Rio Hortega, Valladolid, Spain; 3 Jefe de Servicio de Farmacia, Hospital Universitario La Fe, Valencia, Spain

**Keywords:** electronic device, growth hormone deficiency, treatment adherence, recombinant human growth hormone

## Abstract

**Purpose:** The aim was to identify factors affecting treatment adherence and to assess the clinical, economic and management impact of growth hormone deficiency treatment using an electronic auto-injector for recombinant human growth hormone (r-hGH) administration in children.

**Patients and Methods:** A literature review was conducted in PubMed up to 31JUL2013, including the following search terms: “growth hormone deficiency”, “human-recombinant growth hormone” and “treatment adherence”. An economic model was developed to estimate the economic benefits of using an electronic injection device. In order to quantify this benefit, potential savings due to growth hormone cartridge optimization were analyzed.

**Results:** From the literature review, the following key factors were found to affect treatment adherence: type of device used, discomfort, complexity of treatment regimens, long-term treatment, age and patient or family understanding of treatment benefits were assessed. A better adjustment to prescribed daily dose (accuracy up to 0.01 mg) with the electronic device results in a better optimization of vials and could save an average of 5% of total treatment costs in terms of doses not wasted, amounting to €245 of potential savings per patient and year of treatment.

**Conclusion:** The use of an electronic device for r-hGH administration and monitoring may provide a better identification of responder and adherent patients. It may also generate savings in annual r-hGH consumption by hospitals and regional healthcare services.

## 1. Background

Growth hormone deficiency[1] (GHD) is a relatively uncommon cause of growth retardation and short stature.[2] The prevalence of childhood GHD reported in the literature diverges widely varying between 1/3,480 and 1/30,000 children.[2] Indeed, the incidence of GHD is thought to vary substantially between countries.[3] The Child Growth Foundation estimates that idiopathic GHD occurs in about 1 in 3,800 births in England and Wales.[1] Figures for Belgian population, estimate an overall prevalence of GHD of 1 in 5,600.[2] A Danish study calculated an average incidence rate of childhood-onset GHD of 2.51 per 100,000 population and observed a statistically significant higher incidence rate in males when compared with females.[4] In the United States, approximately 1 in 3,500 children are diagnosed with GHD.[5]

Recombinant human growth hormone (r-hGH) has been found to be an effective treatment for children with GHD and is also indicated for those born small for gestational age or having Turner's syndrome or chronic-renal failure.[6] Multiple preparations of GH are available. Overall, there are no observable differences in the results obtained among the different preparations as long as the regimen follows currently approved daily injections.[7] Long-term GH replacement therapy is recommended by the GH Research Society[8] and the European Society of Paediatric Endocrinology,[9] starting at the time of diagnosis (typically from childhood throughout adolescence and into adulthood), and in Spain criteria for the rational use of GH were officially adopted in 2008.[10] Early intervention with r-hGH treatment results in normal height during childhood and normal or near-normal adult height.[11] Daily injection or needle-free administration is required, and treatment must be sustained over a prolonged period.[12]

Poor adherence to medical treatment is one of the main reasons why patients do not achieve the full benefits of their therapy. It also has a substantial financial weight in terms of money wasted for unused medication and increased healthcare costs including hospitalization due to lack of effectiveness.[13] Indeed, reduced adherence is officially considered a serious economic problem (about 1% of national drug expenditures) that can be mediated through improved administration devices.[14]

Increasing evidence suggests that poor adherence is quite common among children and adults treated with GH.[15] Problems with adherence may be exacerbated by treatment duration; it has been found that treatment duration is inversely related to treatment adherence in chronic diseases.[16]

This study aimed to identify factors affecting treatment adherence and to assess the economic impact of GHD treatment using an electronic auto-injector for r-hGH administration in children.

## 2. Material and Methods

*Literature Review*

A literature review was conducted in PubMed to determine most common problems associated to adherence to GH therapy. Articles published in English or Spanish until 31JUL2013 were retrieved using the search terms: "growth hormone deficiency"; "human-recombinant growth hormone"; "treatment adherence". Articles identified through reference lists and bibliographic searches were also considered.

This review considered studies that included pediatric patients (age 0 to 18 years) with idiopathic GHD, studies assessing treatment adherence and factors that affect it. Exclusion criteria were those studies not carried out in humans or those carried out in adults, those where participants did not have short stature due to GHD, studies in which growth hormone was not administered and those studies where increase in height was not measured as a result.

*Assumptions for the Economic Model*

An economic model was developed to estimate the economic impact related to r-hGH administration using different devices, for a cohort of 100 patients, from the Spanish Healthcare System perspective. There are a variety of presentations of r-hGH marketed in Spain which can be administered using different device types (injector pens, syringes, needle-free and electronic devices), all of them priced at €17.50/mg. For all calculations performed in this evaluation, the smallest vial size available for each presentation of r-hGH was considered.

A model was performed to calculate the annual costs of GH wasted per patient, based on r-hGH cartridge optimization according to the device type used.

The following assumptions were made in the model:

1. The model considered a cohort of pediatric patients aged 4 to 18 years with a diagnosis of idiopathic GHD treated with r-hGH in Spain.[17] Gender and weight distribution were estimated from the literature.[18,19] Table 2 summarizes characteristics of this cohort.
2. Daily dose distribution (mg/kg) among patients in the cohort was estimated considering patient's body weight distribution and the recommended dose for idiopathic GHD treatment. According to summary product characteristics, the recommended dose range lies between 0.025 and 0.035mg/kg/day. The analysis assumed a dose of 0.035mg/kg/day. The model did not take into account possible body weight variations with time.
3. To estimate treatment costs, daily dose (mg) was multiplied by the percentage of patients who required that dose during the time period considered (days) and taking into account the cost per milligram, expressed as ex-factory price. The price of each drug is different depending on the content of somatotropin of each presentation, although the cost per milligram of GH for all preparations available in Spain is €17.50.[20]
4. It was assumed that if the remaining quantity of hormone left in the device after its use was less than 75% of a daily dose, this amount would be discarded. Otherwise, the model assumes a complete cartridge optimization. This model provides results in terms of cost per milligrams wasted per patient and year of treatment, as well as the percentage over total cost.
5. An adherence rate of 100% is assumed for all cases. This rate could be adjusted in the future once the real adherence rate for each patient can be ascertained.
6. The base case considered a time horizon of 12 months and the health care system perspective was taken.

**Table 1. Cohort Age and Weight Distribution (by gender)**

| | % Patients | | | Weight (Kg) | |
|---|---|---|---|---|---|
| **Age** | **N=671** | **Male (54%)** | **Female (46%)** | **Males** | **Females** |
| 4 | 5,06% | 2,73% | 2,33% | 13,00 | 8,88 |
| 5 | 5,06% | 2,73% | 2,33% | 13,55 | 9,25 |
| 6 | 5,06% | 2,73% | 2,33% | 14,14 | 10,27 |
| 7 | 8,09% | 4,37% | 3,72% | 14,75 | 11,86 |
| 8 | 8,09% | 4,37% | 3,72% | 15,64 | 13,92 |
| 9 | 8,09% | 4,37% | 3,72% | 16,99 | 16,36 |
| 10 | 12,46% | 6,73% | 5,73% | 18,94 | 19,07 |
| 11 | 12,46% | 6,73% | 5,73% | 21,51 | 21,96 |
| 12 | 12,46% | 6,73% | 5,73% | 24,69 | 24,94 |
| 13 | 7,30% | 3,94% | 3,36% | 28,37 | 27,90 |
| 14 | 7,30% | 3,94% | 3,36% | 32,40 | 30,76 |
| 15 | 7,30% | 3,94% | 3,36% | 36,52 | 33,41 |
| 16 | 0,42% | 0,23% | 0,19% | 40,43 | 35,77 |
| 17 | 0,42% | 0,23% | 0,19% | 43,75 | 37,73 |
| 18 | 0,42% | 0,23% | 0,19% | 46,03 | 39,21 |

## 3. Results

*Search Strategy*

Sixty-one references were identified by our bibliographic search strategy. In addition, 36 references were found after a manual reference search from relevant articles from the bibliographic search strategy.

*Literature Review: Clinical Benefits*

Articles included in this review indirectly assessed treatment adherence based on questionnaires administered both to children with GHD and their parents. Key factors affecting treatment adherence included, amongst others: discomfort (related to daily injections), complexity of treatment regimens (daily dose required), long-term treatment, age and patient or family understanding of treatment benefits and effects of lack of adherence. (See Table 1)

Patient's low motivation may further compromise treatment adherence, given that the therapeutic benefits are not immediate.[33] There is also some evidence that, at least in the short term, patient choice is associated with improved adherence which in turn is associated with improved height velocity.[29] Furthermore, injectable therapies are perceived as painful and difficult to administrate which may hamper treatment compliance.[16] Poor adherence and persistence can have a major negative impact on the long-term clinical effectiveness of r-hGH treatment. A study measuring compliance found that over two-thirds of patients were not adequately complying with therapy.[28] Patients who missed more than half of their monthly dose had a lower annual growth (6.3 cm per year) than those who missed less than half of their doses (8.9-9.1 cm per year).[26] Thus, accurate monitoring of adherence rates is important, as it enables poor adherence to be detected and acted upon.[12] (See Figure 1)

**Table 2. Important Aspects to Consider in Adherence to Growth Hormone Therapy**

| Author | Publication Year | Sample Studied | Variable(s) to Measure Adherence and/or Compliance | Evaluation Type | Areas Evaluated |
|---|---|---|---|---|---|
| **Gacs, et al (21)** | 1991 | 78 patients | Parent´s educational level | Medical history; Questionnaires | Socioeconomic |
| **Lieberman, et al (22)** | 1993 | 96 patients | Degree of treatment satisfaction | Questionnaire with 50 items | Child´s psychological surrounding; Perception of medical problem; Satisfaction with medical treatment and results achieved |
| **Smith, et al (23)** | 1995 | 177 patients | Understanding of prescribed r-GH treatment; Evaluation of number of doses missed | Questionnaire with 22 items | Cognitive |
| **Lopez-Siguero, et al (24)** | 1995 | 90 patients | Understanding of prescribed r-GH treatment; Knowledge of pathology; Acceptance of therapy | Questionnaire with 21 items | Child´s psychological surrounding; Cognitive |
| **Oyarzabal, et al (25)** | 1998 | 473 patients | Evaluation of number of doses missed; Type of device used; Understanding of prescribed r-GH treatment | Questionnaire with 28 items | Cognitive; Technological innovation |
| **Desrosiers, et al (26)** | 2005 | 631 patients | Type of device used (Electronic, with needle, without needle) | Questionnaire | Technological innovation |
| **Wickramasuriya, et al (27)** | 2006 | 125 patients | Type of device used; Factors influencing choice of device | Questionnaire | Technological innovation |
| **Haverkamp, et al (15)** | 2008 | | Type of device used; Understanding of prescribed r-GH treatment; Doctor-patient relationship | Bibliographic review | Technological innovation; Cognitive |
| **Rosenfeld, et al (28)** | 2008 | 724 patients | Understanding of prescribed r-GH treatment; Training and r-GH administration technique; Doctor-patient relationship; Motivation, acceptance and satisfaction with therapy; Type of device used | Questionnaire with134 items | Cognitive; Emotional / Psychological; Technological innovation |
| **Kapoor, et al (29)** | 2008 | 75 patients | Treatment duration; Type of device used; Educational level of parents and family; Duration of medical consultation; Type of health profesional providing patient training | Questionnaires | Technological innovation; Socioeconomic; Psychological; Educational |
| **Norgren, et al (30)** | 2009 | | Understanding of prescribed r-GH treatment; Acceptance of therapy; Acceptance of type of device used; Acceptance of type of device used; Acceptance of results obtained; Doctor-patient relationship; Motivation, acceptance and satisfaction with therapy; Motivation, acceptance and satisfaction with therapy; Family setting; Educational level; Training and r-GH administration technique; Age | Bibliographic review | Cognitive; Psychological/ emotional; Socioeconomic level |
| **Fuchs, et al (16)** | 2009 | 70 patients | Type of device used (ease of use); Acceptance of device used | Questionnaire with 21 items | Technological innovation; Psychological |
| **Pfutzner, et al (31)** | 2010 | 56 patients | Type of device used (ease of use); Acceptance of device used | Questionnaire with 21 items | Technological innovation |
| **Bozzola, et al (12)** | 2011 | 824 patients | Type of device used (ease of use); Acceptance of device used | Data registered in electronic device; Questionnaire of 15 items | Technological innovation |
| **Kappelgaard, et al (32)** | 2011 | 74 patients | Type of device used (ease of use); Acceptance of device used | Questionnaire with 26 items | Technological innovation |

r-hGH: Recombinant human growth hormone

**Figure 1. Diagram for Growth Hormone Treatment Outcomes Optimization**

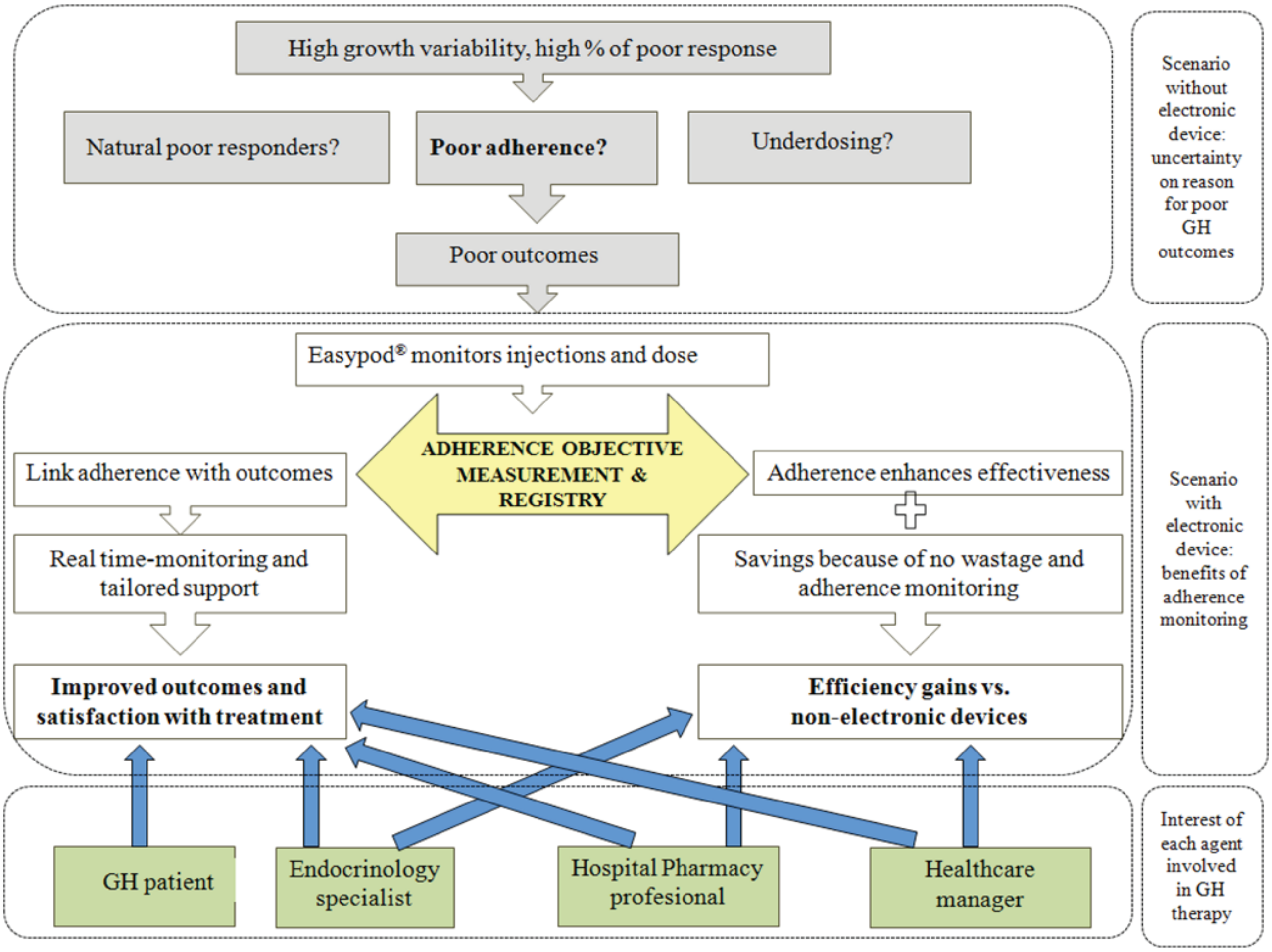

GH: growth hormone

Another main factor associated with poor adherence was the underestimation of the consequences of missing doses, which has been reported in several studies.[11, 28, 30] In this sense, these studies pointed out the relevance of good education strategies for patients, parents and family members as a way to improve adherence.

There has been considerable technological progress in the methods of administering r-hGH in recent years, which have been developed to optimize treatment and improve patient's experience with daily injections.[34] The development of devices providing more accurate and objective data has allowed clinicians to perform real assessments of patient's response and adherence to treatment. In this sense, there is one electronic device on the market (Easypod™) that monitors when the doses of r-hGH are taken and records the data for future evaluation. The device digitally displays the dose that has been pre-programmed by the physician, the dose administered, the amount remaining within the device, the date of the last injection and the injection history. By recording and storing treatment details, the physician can assess whether doses are being taken as prescribed.[11]

*Literature Review: Management Benefits*

Electronic recording of r-hGH dosing history may also lead to management benefits such as including adherence in reimbursement agreements.[13] Outcomes-based agreements or risk-sharing agreements where financial outcomes are subject to an objective measurement of patient adherence may incentivize healthcare providers to address poor adherence.[13] Under such schemes, every agent involved in r-hGH therapy (patient, Endocrinology Specialist, hospital pharmacy, healthcare manager) would perceive the clinical (effectiveness) and economic (lower direct and indirect cost of compliant patients vs. non-compliant patients[14]) benefits of adherence.

Another source of value for healthcare professionals and managers gained by electronic recording is the feasibility of assessing clinical and economic efficiency of resources devoted to r-hGH therapies by correlating adherence, outcomes and consumption variables. It would ease the task of researching the impact of poor adherence on effectiveness variables such as height velocity and linear growth.[29,38]

*Economic Evaluation: Economic Benefits*

From the literature review, we were able to determine the most important variables affecting GH treatment, which allowed us to develop an economic model based on the assumptions described in the Material and Methods section. Mean age and weight for patients included in the assessed cohort was 10 years and 21.5 kg. The mean dose of GH administered for the patients in the assessed cohort was 0.77 mg per day. According to cohort characteristics, mean annual treatment costs were estimated at €4,806 per patient. Dosage distribution among patients in the cohort and annual treatment cost are described in Figure 2.

**Figure 2. r-hGH Dosage (milligrams per day) Distribution in the Cohort**

r-hGH: Recombinant human growth hormone

A better adjustment to prescribed daily dose (accuracy up to 0.01 mg as indicated by the manufacturer) by the electronic injection device leads to a better optimization of vials. Drug wastage is also minimized as, when the dose remaining in the previous cartridge is not enough to self-administer the daily dose, a partial dose can be injected with one cartridge and a second injection can make up the complete daily dose.[37]

Estimated costs due to GH wasted ranged between €100 and €356 per patient and year of treatment for pen and needle-free injection devices, respectively. Hormone wastage for the electronic device was €0 as it performs an automatic daily dose adjustment according to the cartridge volume. For the different injection devices assessed these costs represented between 2.11% and 7.53% of total treatment costs.

The use of an electronic device for GH administration could save an average of 5% of total treatment costs only in terms of doses not wasted, amounting to €245 of potential savings per patient and year of treatment. The device, as well as all disposable material (such as needles), and training in the use of the device is provided to patients free of charge (as indicated by electronic device manufacturer).

Another source of economic benefits would result from adjusting monthly or bimonthly therapy dispensation according to actually self-administered milligrams as recorded by the electronic device. This practice may lead to a better adjustment of r-hGH vials funded by hospital pharmacy service. The only published mean adherence to r-hGH treatment using the electronic device, Easypod™, was 80% (range 73%- 84%) in a cohort of 87 patients whose mean age was 11.1 years.[35] This study concludes that 'this reliable monitoring of patient adherence is the first step towards optimal management of adherence issues'. If we assume that average adherence in Spanish r-hGH patients reaches 80%, adjusting therapy dispensation would lead to potential savings up to 20% of annual drug cost. This potential saving could be even larger if we consider a recent review that estimates GH adherence between 36% and 49%.[15]

## 4. Discussion

R-hGH is an effective treatment for children with GHD. However, eligibility for treatment with GH is one of the most controversial areas in pediatric endocrinology. Members of the Lawson Wilkins Pediatric Endocrinology Society cited the cost as one major factor limiting the prescription of the drug.[36] In this sense, the use of an electronic device could allow a reduction of r-hGH therapy costs. The device allows an automatic dose adjustment per cartridge (minimizing drug wastage), and may be used for monitoring adherence. In this sense, our study demonstrates that r-hGH administration by the electronic device assessed could reduce therapy costs by an average of 5% by minimizing wastage and, it is possible that, monitoring adherence could reduce these costs even further.

Assessing long-term adherence and persistence to treatment has always been difficult.[37] Different methods for following adherence, ranging from self-reporting to direct observation by a healthcare professional, vary in reliability, ease of use and cost.[30] Direct questioning of a patient on adherence can result in over-estimation of adherence, particularly when the patient wishes to please the physician.[11] Cutfield *et al.* indicate that patient and/or parent reported compliance is an unreliable measure of GH treatment compliance.[38]

A recent review on GH treatment for children concludes that the awareness, recognition and management of poor response to growth hormone treatment is bound to lead to better patient care, a greater cost-effectiveness and increased opportunities for clinical benefit.[39] As shown in a recently published systematic review,[40] analyzing the causes of non-adherence is complicated, but we believe a complete assessment of adherence should encompass a measure of whether the correct dose of GH

has been taken and whether consecutive doses have been taken at the correct interval. Direct observation of GH levels or its metabolites, provides good data, but it is impractical, as these tests are costly and inconvenient for patients. Also, as patients have to provide blood samples, testing is more likely to increase adherence, so results may not be representing real practice. The use of indirect methods such as self-reporting, cartridge accountability, and rate of prescription refills could overcome the limitations of direct methods. Electronic devices provide a log of treatment doses and timings, making it possible to identify the periods or occasions when doses are missed, without compromising the convenience to patients. In this sense, Bang *et al.* suggest that adherence may improve with the use of GH injection devices that include computerized pen systems with skin sensors documenting the dose and frequency of injections.[39] An objective monitoring of treatment adherence will also help physicians to identify non-responder patients from non-adherent patients. Monitoring real adherence could also help resolve doubts relating to the need for a dose increase as well as the need for additional education of the patient. It is also crucial for treatment success to be certain that patients are receiving the correct dose. The use of an electronic device for injection could avoid patient errors, as the dose is set by the doctor or nurse, preventing patients from manipulating the device. This feature could prevent under-dosing, which could decrease clinical effectiveness and final treatment outcomes, as well as overdosing, which generates additional costs for healthcare systems and could be avoided with more accurate dosage adjustments.

Studies of other chronic conditions, have consistently found significant reductions in health care costs associated with interventions to improve adherence, such as a study that estimated the savings produced by optimal control of severe asthma to be approximately 45% of the total medical costs.[14]

Our study has highlighted the clinical, economic and management benefits associated with the use of an electronic injection device for GH administration. The results of our analysis demonstrate that r-hGH administration using this kind of device could help optimize economic resources for health care providers as well as improving management of treatment adherence. Agenda for further research may focus on potential benefits of self-administration of GH with an electronic device in adult GH deficiency patients.

## 5. Conclusions

The use of an electronic injection device yields clinical, economic and management benefits for both patients and healthcare professionals, which are summarized in Table 3. This form of administration allows:

a) A more efficient use of drug dispensed by minimizing waste between administrations, could allow a savings of over 7% of the cost of the drug.

b) A reliable and simple follow-up of patient´s adherence to treatment and consequently, a precise assessment of their clinical results.

c) A perfect identification of patients that, despite strictly following their treatment regimen, do not respond to it. Notwithstanding, the device is a "technological marker" for prediction of response or identification of non-responder patients.

Economic analysis of available GH should, therefore, not only consider the price of the hormone, as there are other considerations, dose optimization across cartridges and adherence monitoring, that could lead to better effectiveness and efficiency outcomes with the concomitant benefits for patients and health services.

**Table 3. Review of Benefits associated with Self-administration of r-hGH with an Electronic Device**

| CATEGORY | BENEFIT |
|---|---|
| CLINICAL | ✓ Identifying the patient profile and reason for poor outcomes:<br>- Responder vs. natural poor responder to r-hGH<br>- Highly compliant vs. lowly compliant |
| | ✓ Moving towards personalized medicine and tailored support (biophychosocial strategies) |
| | ✓ Features that may foster patient compliance: customizable injection setting (needle speed, injection speed, injection time and injection depth), automatic needle attachment, skin sensor, audible and visual signals. |
| ECONOMIC | ✓ Better adjustment to prescribed daily dose (accuracy up to 0.01 mg) |
| | ✓ Absence of drug wastage between administrations (annual savings of up to 7%) |
| | ✓ Adjustment of drug dispensation to self-administered mg (annual savings of up to 20%) |
| MANAGEMENT | ✓ Outcomes-based agreements to incentivize adherence improvements |
| | ✓ Assessment of clinical and economic efficiency of r-hGH therapies |

r-hGH: Recombinant human growth hormone

## Declaration of Competing Interests

The study was funded by a grant from Merck Quimica S.L., to the Health Institute Carlos III, Agencia de Evaluación de Tecnologías Sanitarias, of which Z. Saz-Parkinson, M. Granados, C. Bouza and J.M. Amate are employees.
